# Supplementary material for: Blood-brain barrier opening with focused ultrasound in Parkinson’s disease dementia
Source: Nat Commun. 2021 Feb 3;12:779. doi: 10.1038/s41467-021-21022-9 (PMC7859400; doi:10.1038/s41467-021-21022-9)
Supplement: Supplementary file 1 — Supplementary Material [file 41467_2021_21022_MOESM1_ESM.pdf]

**SUPPLEMENTARY MATERIAL**

**Supplementary table 1.** Sonication parameters.

| | STAGE 1 | | | | | STAGE 2 | | | | |
|---|---|---|---|---|---|---|---|---|---|---|
| | Min power (W) | Max power (W) | Average sonication time | Total of sonications | Total volume (mm3) | Min power (W) | Max power (W) | Average sonication time | Total of sonications | Total volume (mm$^3$) |
| **Patient 1** | 5 | 40 | 90 | 8 | 46 | 26 | 60 | 90 | 8 | 160 |
| **Patient 2** | 6 | 8 | 90 | 2 | 50 | 5 | 6 | 90 | 2 | 40 |
| **Patient 3** | 6 | 7 | 90 | 3 | 50 | 4 | 6 | 76 | 5 | 30 |
| **Patient 4** | 12 | 16 | 71 | 5 | 20 | 8 | 11 | 69 | 7 | 60 |
| **Patient 5** | 6 | 14 | 88 | 8 | NA | 10 | 14 | 77 | 7 | NA |
| **Mean** | 7 | 17 | 85.8 | 5.2 | 41.5 | 10.6 | 19.4 | 80.4 | 5.8 | 72.5 |
| **SD** | 2,8 | 13,4 | 8.3 | 2.8 | 14.5 | 8.9 | 22.9 | 9.3 | 2.4 | 59.7 |

**Supplementary table 2.** Motor scores at baseline and post BBB opening procedures.

| Motor scores | | Baseline | Follow up |
|---|---|---|---|
| MDS-UPDRS | I | 20 (8.83) | 19.8 (8.07) |
| | II | 24.4 (11.89) | 24.2 (11.12) |
| | III | 53.2 (10.8) | 49.8 (13.1) |
| | IV | 1.4 (1.95) | 1.2 (1.6) |

Follow up motor evaluation was performed 3 weeks after the stage II treatment. MDS-UPDRS: Movement Disorder Society-sponsored revision of the Unified Parkinson's Disease Rating Scale part III. PD: Parkinson´s disease. SD: Standard deviation.

**Supplementary table 3**. [18F]-Flutemetamol PET/MR scans results

Quantitative standardized uptake value ratio (SUVr) and percent change in SUVr in the regions of interest containing sonication volumes on [18F]-flutemetamol PET/MR scans. SUVr was calculated by normalizing to the pons.

| | **Baseline** | **Post-treatment** | **% Change** |
|---|---|---|---|
| Patient 1 | 0.68 | 0.66 | -0.02 |
| Patient 2 | 0.55 | 0.53 | -0.05 |
| Patient 3 | 1.09 | 1.40 | 0.28 |
| Patient 4 | 0.82 | 0.92 | 0.11 |
| Patient 5 | 0.80 | 0.85 | 0.06 |
| **Mean (SD)** | **0.79 (0.20)** | **0.87 (0.33)** | **0.08 (0.13)** |

**Supplementary table 4.** [18F]-FDG PET/MR scans results

Quantitative standardized uptake value ratio (SUVr) and percent change in SUVr in the regions of interest containing sonication volumes on [18F]-FDG PET/MR scans. SUVr was calculated by normalizing to the pons.

| | **Baseline** | **Post-treatment** | **% Change** |
|---|---|---|---|
| Patient 1 | 0.84 | 0.97 | 0.16 |
| Patient 2 | 1.46 | 1.23 | -0.16 |
| Patient 3 | 0.98 | 0.96 | -0.03 |
| Patient 4 | 1.17 | 1.03 | -0.12 |
| Patient 5 | 0.97 | 0.95 | -0.03 |
| **Mean (SD)** | **1.08 (0.24)** | **1.03 (0.12)** | **-0.03 (0.12)** |

**Supplementary table 5.** Inclusion and exclusion criteria

<u>Inclusion criteria</u>

1. Age: 60-80 years
2. Parkinson´s Disease and mild-moderate dementia according to the Movement Disorders Criteria
3. Able and willing to give informed consent or has delegated this to a substitute decision maker.
4. Mini Mental State Exam (MMSE) scores ≥16 (able to complete a Neuropsychological evaluation)
5. Geriatric Depression Scale (GDS) score ≤20
6. If receiving concurrent treatment with an AChEI and/or memantine, has been on the medication for at least 4 months with a stable dose for at least 3 months
7. Neuroimaging abnormalities (hypometabolism, amyloid deposition, and/or atrophy) in the proposed target region: right parietooccipitotemporal cortex

8. Able to communicate sensations during the ExAblate® MRgFUS procedure
9. American Society of Anesthesiologists Physical Status Classification System (ASA) <3
10. Able to attend all study visits (i.e., life expectancy of ≥1 year).

Exclusion criteria

1. Contraindications to MRI, MRI contrast, or ultrasound contrast
2. MRI findings of active or acute neurological process (e.g. infection, space occupying lesion) or macrohemorrhage or >4 lobar microbleeds
3. No more than 1 non-strategic lacune <1.5 cm
4. Known cerebral or systemic vasculopathy
5. More than 30% of the skull area traversed by the sonication pathway is covered

by scars, scalp disorders, or atrophy of the scalp

6. Clips or other metallic implanted objects in the skull or the brain, except shunts
7. Significant cardiac disease or unstable hemodynamic status
8. Uncontrolled hypertension
9. Predisposition to bleeding
10. Significant depression and at potential risk of suicide
11. Impaired renal function
12. Severe chronic respiratory disorders
13. Does not have a reliable caregiver in frequent contact with the patient and who can accompany the patient to the clinic and treatment or be available by telephone at designated times. Participants living in retirement homes may be included. Caregiver not willing to sign the Informed Consent Form

14. Currently in a clinical trial involving an investigational product or non-approved use of a drug or device or in any other type of medical research
15. History of seizure disorder or epilepsy
16. History of drug or alcohol use disorder
17. Presence of potential blood-born infections

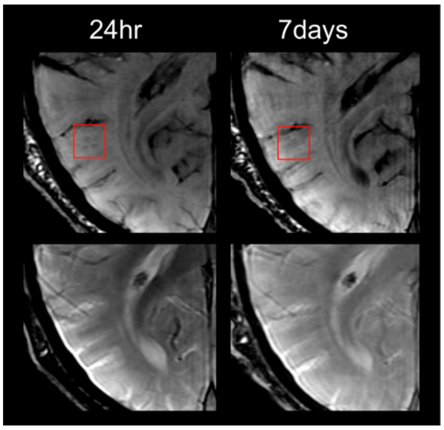


**Supplementary figure 1.** T2*-weighted and SWAN MRI findings in patient 3

Susceptibility weighted angiography (SWAN) (upper line) and T2* (lower line) images in patient 3, stage 1 treatment. SWAN images show discrete round hypointensity in the sonicated region 24 hours after sonication which disappeared within one week in one week. T2* images show no abnormalities.

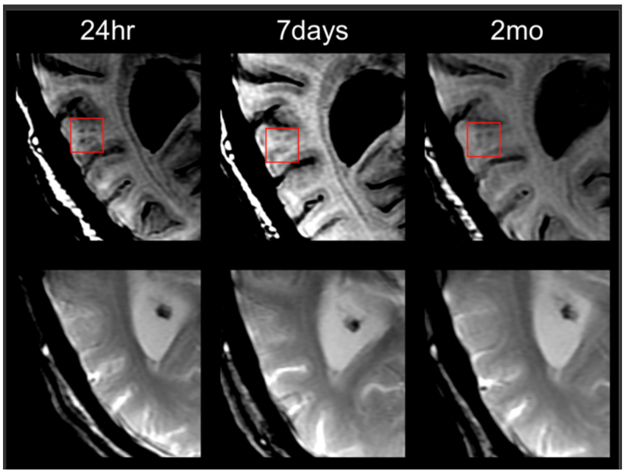


**Supplementary figure 2**. T2*-weighted and SWAN MRI findings in patient 2

Susceptibility weighted angiography (SWAN) (upper line) and T2* (lower line) images in patient 2. SWAN images show discrete round hypointensity in the sonicated region 24 hours after sonication, stage 1 treatment. These progressively decreased and were only minor after 2 months of stage 2 treatment. T2* images show no abnormalities.

mo= months
