# Supplementary material for: Blood-brain barrier opening with focused ultrasound in Parkinson’s disease dementia
Source: Nat Commun. 2021 Feb 3;12:779. doi: 10.1038/s41467-021-21022-9 (PMC7859400; doi:10.1038/s41467-021-21022-9)
Supplement: Supplementary file 3 — Reporting Summary [file 41467_2021_21022_MOESM3_ESM.pdf]

natureresearch

Corresponding author(s): José A Obeso

Last updated by author(s): 2020/06/10

Reporting Summary

Nature Research wishes to improve the reproducibility of the work that we publish. This form provides structure for consistency and transparency in reporting. For further information on Nature Research policies, see Authors & Referees and the Editorial Policy Checklist .

Statistics

For all statistical analyses, confirm that the following items are present in the figure legend, table legend, main text, or Methods section.

n/a	Confirmed	
x	☐	The exact sample size (*n*) for each experimental group/condition, given as a discrete number and unit of measurement
x	☐	A statement on whether measurements were taken from distinct samples or whether the same sample was measured repeatedly
x	☐	The statistical test(s) used AND whether they are one- or two-sided<br>*Only common tests should be described solely by name; describe more complex techniques in the Methods section.*
x	☐	A description of all covariates tested
x	☐	A description of any assumptions or corrections, such as tests of normality and adjustment for multiple comparisons
x	☐	A full description of the statistical parameters including central tendency (e.g. means) or other basic estimates (e.g. regression coefficient) AND variation (e.g. standard deviation) or associated estimates of uncertainty (e.g. confidence intervals)
x	☐	For null hypothesis testing, the test statistic (e.g. *F*, *t*, *r*) with confidence intervals, effect sizes, degrees of freedom and *P* value noted<br>*Give P values as exact values whenever suitable.*
x	☐	For Bayesian analysis, information on the choice of priors and Markov chain Monte Carlo settings
x	☐	For hierarchical and complex designs, identification of the appropriate level for tests and full reporting of outcomes
x	☐	Estimates of effect sizes (e.g. Cohen's *d*, Pearson's *r*), indicating how they were calculated

Our web collection on statistics for biologists contains articles on many of the points above.

Software and code

Policy information about availability of computer code

Data collection	Clinical and neuroimaging data were acquired. Not specific software was appliead
Data analysis	SPM 12 for PET analysis (amyloid and fluorodeoxyglucose). ITK-Snap segmentation software version 3.8.0. for segmentation.

For manuscripts utilizing custom algorithms or software that are central to the research but not yet described in published literature, software must be made available to editors/reviewers. We strongly encourage code deposition in a community repository (e.g. GitHub). See the Nature Research guidelines for submitting code & software for further information.

Data

Policy information about availability of data

All manuscripts must include a data availability statement. This statement should provide the following information, where applicable:

- Accession codes, unique identifiers, or web links for publicly available datasets
- A list of figures that have associated raw data
- A description of any restrictions on data availability

The data that support the findings of this study are available from the corresponding author upon reasonable request.

Field-specific reporting

Please select the one below that is the best fit for your research. If you are not sure, read the appropriate sections before making your selection.

☒ Life sciences ☐ Behavioural & social sciences ☐ Ecological, evolutionary & environmental sciences

For a reference copy of the document with all sections, see nature.com/documents/nr-reporting-summary-flat.pdf

Life sciences study design

All studies must disclose on these points even when the disclosure is negative.

Sample size	No statistical methods were used to predetermine sample size because this is an open, proof-of-concept, safety and feasibility pilot study. We decided to report 5 patients because that is thought sufficient for initial estimation of viability and safety and similar to other articles in other neurodegenerative diseases.
Data exclusions	All adverse events were included, based on previous treatment experience with MRgFUS. Adverse events were reported following the pre-stablished conditions listed on the protcol: device related, Expected procedure findings, Drug/Contrast media reactions, Medical conditions, and Unrelated to device or procedure. Some anecdotal events were not included.
Replication	Reproducibility was verified by the repetition of blood brain barrier opening procedure in all patients, the treatment was repeated a second time, with successfull BBB opening
Randomization	There was no randomization, since it was a pilot exploratory study. Therefore, CONSORT that stands for clinical trials does not apply here.
Blinding	Investigators were not blinded, it was a feasibility and safety study

Reporting for specific materials, systems and methods

We require information from authors about some types of materials, experimental systems and methods used in many studies. Here, indicate whether each material, system or method listed is relevant to your study. If you are not sure if a list item applies to your research, read the appropriate section before selecting a response.

Materials & experimental systems

n/a	Involved in the study	
☒	☐	Antibodies
☒	☐	Eukaryotic cell lines
☒	☐	Palaeontology
☒	☐	Animals and other organisms
☐	☒	Human research participants
☐	☒	Clinical data

Methods

n/a	Involved in the study	
☒	☐	ChIP-seq
☒	☐	Flow cytometry
☐	☒	MRI-based neuroimaging

Human research participants

Policy information about studies involving human research participants

Population characteristics	Parkinson´s disease patients between ages 60 and 80 years with mild-to-moderate dementia, without severe depression, able to attend all study visits and lack of systemic disease (i.e. significant cardiovascular disease , severe cerebrovascular ischemia, uncontrolled hypertension, impaired renal function, severe chronic respiratory disorders). All the recruited patients were male.
Recruitment	All subjects evaluated in the clinic that met inclusion criteria were informed about the study. We screened out and discussed the possibility of entering this study in 16 patients and overall, there has been a greater male representation. This has ocurred involuntarily regarding the investigators and probably reflects availablity and williness to enter in research study, but fundamenally and mainly that we are dealing with a low N of subjects. Surely, a greater number of patients would lead to a more even gender ratio.
Ethics oversight	the Research Ethics Board at HM Hospitales 17.07.1101-GHM and the Spanish Agency of Medicines and Medical Products number 627/17/EC

Note that full information on the approval of the study protocol must also be provided in the manuscript.

Clinical data

Policy information about clinical studies

All manuscripts should comply with the ICMJEguidelines for publication of clinical research and a completedCONSORT checklist must be included with all submissions.

Clinical trial registration	NCT03608553
Study protocol	The study protocol is available on the suplelemtary information.
Data collection	Patient recruitment took place at the Movement Disorders outpatient clinic (CINAC-HM Puerta del Sur) and by refererals from other Madrid region specialized unit. It started in october 2018 and ended in april 2019

Outcomes	The primary outcome measures were safety and feasibility, predetermined according to a phase I pilot study. Safety was assessed by neurological and neuropsychological examination and MR imaging. The feasibility of reversible and repeated BBB opening was determined by contrast enhancement in the target regions with resolution within the following week. The other outcomes (changes in amyloid and fluorodeoxyglucose PET) were considered secondary and analyzed using Statistical Parametric Mapping (SPM12).

Magnetic resonance imaging

Experimental design

Design type	MRImages were acquired during the procedure following the parameter described below.
Design specifications	No fMRI was carried out, only structural
Behavioral performance measures	NA

Acquisition

Imaging type(s)	Structural
Field strength	3T
Sequence & imaging parameters	Brain MRI protocol was performed on a 3T GE scanner (Discovery 750w, GE Healthcare, Milwaukee, WI) including: 3D Fast-Spoiled Gradient Recalled Echo T1-weighted [TR/TE/TI 9352/4.02/450ms, flip-angle 10º, FoV 256mm, acquisition matrix 256x256, slice thickness 1mm], axial Fast Spin Echo T2-weighted [TR/TE 8093/94.08ms, flip-angle 111º, FoV 256mm, acquisition matrix 320x320, slice thickness 2mm], axial Gradient Echo T2*-weighted [TR/TE 680/18ms, flip-angle 15º, FoV 256mm, acquisition matrix 448*224, reconstruction matrix 512x512, slice thickness 6mm], axial Fluid Attenuated Inversion Recovery (FLAIR) T2-weighted [TR/TE/TI 9000/141/2468ms, flip-angle 170º, FoV 256mm, acquisition matrix 320x224, reconstruction matrix 512x512, slice thickness 6mm], axial spin-echo EPI diffusion weighted [TR/TE 8000/78.2ms, flip-angle 90º, FoV 240mm, acquisition matrix 140x140, reconstruction matrix 240x240, slice thickness 4.4mm, NEX 2, bvalue = 1000, single encoding direction] and axial susceptibility-weighted images (SWI) [TR/TE 56.7/23.176ms, flip-angle 30º, FoV 220mm, acquisition matrix 384x256, slice thickness 2mm].
Area of acquisition	Whole brain coverage including cerebellum.

Diffusion MRI ☐ Used ☒ Not used

Preprocessing

Preprocessing software	There was no processing of the images acquired during the BBB opening procedure, but a manual segementation with ITK-Snap segmentation software version 3.8.0.
Normalization	No normalization was used
Normalization template	No normalization
Noise and artifact removal	Not performed
Volume censoring	Not performed

Statistical modeling & inference

Model type and settings	Images acquired during the treatment were not statistically analysed
Effect(s) tested	NA

Specify type of analysis: ☐ Whole brain ☐ ROI-based ☐ Both

Statistic type for inference (See Eklund et al. 2016)	NA
Correction	NA

Models & analysis

n/a	Involved in the study
☒	☐ Functional and/or effective connectivity
☒	☐ Graph analysis
☒	☐ Multivariate modeling or predictive analysis
